# Nanoparticle Lysis of *Cryptosporidium* Oocysts

**DOI:** 10.3390/mps7050066

**Published:** 2024-08-23

**Authors:** Ameya Vaidya, Claire Bankier, Helinor Johnston, Helen Bridle

**Affiliations:** Institute of Biological Chemistry, Biophysics and Bioengineering, Heriot Watt University, Edinburgh EH14 4AS, UK

**Keywords:** nanoparticles, *Cryptosporidium*, lysis, DNA extraction

## Abstract

The extraction of DNA from *Cryptosporidium* oocysts is challenging due to the robust oocyst wall. Nanoparticles have been applied to disinfect *Cryptosporidium* oocysts; here, we demonstrate the utilisation of nanoparticles to disrupt the oocyst wall to enable sporozoite lysis and detection via PCR. Both silver and zinc oxide nanoparticles are investigated under different conditions and compared to existing techniques. Zinc oxide nanoparticles are shown to be as effective as freeze–thaw methods, suggesting that a nanoparticle lysis approach offers a viable alternative to existing methods.

## 1. Introduction

*Cryptosporidium* is a protozoan pathogen. It is associated with waterborne and foodborne transmission, which have considerable human health impacts [1,2,3,4], as well as livestock disease [5,6,7]. Recently, various molecular methods have been developed and employed for detection, outbreak investigation, and subtype identification [5,8].

One of the challenges with the molecular detection of *Cryptosporidium* is the disruption of the oocyst wall to enable the lysis of the internal sporozoites and the release of DNA [9,10,11]. Different techniques have been utilised, including freeze/thaw cycling [12,13], thermal inactivation [14], bead beating [15,16], mechanical disruption, and sonication [17], as well as a combination of these approaches [8,18,19]. It is unclear which is the optimal approach in water, although a recent study compared techniques on stool samples [9]. Freeze/thaw cycling requires access to, and handling facilities for, liquid nitrogen and is a time-consuming process. Bead beating requires relatively expensive equipment. Sonication appears not to be fully effective unless combined with one of the other approaches. A more efficient, low-cost method for oocyst disruption requiring minimal facilities would be useful for more rapid sample processing. Others are developing a surfactant-based extraction technique to achieve this goal [10]; however, we suggest that nanoparticles should be employed to achieve this goal.

Nanoparticles (NP) have been used in disinfection applications for microorganisms, including protozoan pathogens, for a long time [20,21]. Silver nanoparticles (Ag NPs) have previously been used to disrupt the *Cryptosporidium* oocyst wall and results show a significant reduction in viability at concentrations above 0.5 mg/mL, as determined by excystation assays [22], as well as the loss of integrity of the oocyst wall, as confirmed by dielectrophoretic measurements [23]. Ag NPs have been applied for disinfection in both water samples [24] and faecal samples [25]. Chitosan and copper oxide nanoparticles have also been utilised in disinfection studies [26,27], whereas ZnO NPs have been used for disease treatment in mice [28]. A recent review summarises the impact of NPs on protozoan pathogens [21]. Of additional benefit is the fact that NPs are low-cost, with ~1 mg of AgNPs costing less than 5p.

Here, we report on an investigation into the ability of NPs to disrupt and lyse *Cryptosporidium parvum* oocysts, utilising silver NPs (selected as the most widely studied NP with *Cryptosporidium*) and zinc oxide (ZnO) NPs (selected due to their previous use in *Cryptosporidium* treatment [28] and their known effectiveness against other microbes [29,30]) in water samples.

## 2. Materials and Methods

### 2.1. Nanoparticles

Two different NPs were investigated: silver NPs (NM300) and zinc oxide (ZnO) NPs (NM110). Both were obtained from the JRC Nanomaterial Repository (Ispra, Italy) [31]. The anti-microbial properties of these are well established [29,30,32,33]. Dynamic light scattering (DLS) measurements were used to confirm NP size. Briefly, stock suspensions of ZnO and Ag nanoparticles were prepared in filtered (0.2 µm) DI water at a concentration of 1 mg/mL; they were sonicated for 16 min in a bath sonicator and then serially diluted to 1, 5, 10, 20, and 50 µg/mL in DI water. Note that we could not test the exact concentration range tested in the oocyst studies as NP concentrations above 50 µg/mL are not suitable for DLS analysis. After preparation, samples were analysed immediately at 22 °C using a Zetasizer Nano Series, Malvern (Malvern Panalytical Ltd., Malvern, UK).

### 2.2. Protozoa

*Cryptosporidium parvum* oocysts (*Cryptosporidium* Production Laboratory, University of Arizona, Tuscon, AZ, USA) were vortexed (SA8 Stuart Vortex Mixer, Bibby Scientific Ltd., Stone, UK) before dilution in DI water (Milli Q Integral 3, Merck Millipore KGaA, Darmstadt, Germany). Oocyst stocks were prepared through serial dilutions in DI water to obtain oocyst numbers that ranged from 10 and 10,000 (a value of 1000 was used for the exposure time study). 

### 2.3. NP Exposures 

The oocysts were exposed to a NP suspension (200 µL) at concentrations ranging from 0.125 to 1 mg/mL for between 0 to 120 min at room temperature prior to performing the DNA extraction and purification protocol (proteinase K exposure at 56 °C for 1 h, followed by use of a kit, Macherey-Nagel GmBH, Düren, Germany) [12]. No attempt was made to remove the NPs before the DNA extraction and purification steps. There was a concern that the presence of NPs during the amplification phase of the PCR would impact performance; to determine whether there was an impact on the amplification, the PCR kit positive control was run under three conditions: (1) as provided by the manufacturer (Ct = 27.5); (2) spiked with Ag NPs (Ct = 28.0); and (3) spiked with ZnO NPs (Ct = 30.4). The Ct values were not significantly different from each other, unlike in some previous research [34,35], suggesting that there is no impact of these NPs on amplification with the kit used in this study. One further explanation could be that a degree of removal occurs during the DNA extraction and purification protocol; AgNP precipitation on the Eppendorf tube walls was observed (see Figure S1). 

### 2.4. Oocyst Detection 

To detect *Cryptosporidium* DNA, a *Cryptosporidium* probe and primer kit (CeeramTools, SAS Ceeram, Biomerieux, Marcy-l’Étoile, France) was used and samples were run on an Applied Biosystems PCR Machine. Positive controls from the PCR kit containing *Cryptosporidium* DNA (average Ct value of 27.5 (SD = 0.81), Figure S1 were included in every PCR run. The NP-mediated lysis of oocysts was compared against the commonly utilised approach of freeze–thaw, adopting the following protocol: oocysts were exposed to liquid nitrogen (−196 °C; 1 min), before immersion in a heated water bath at 56 °C until they were fully thawed, ten times [12]. The freeze–thaw samples acted as a positive control for the whole process. 

Negative control experiments, i.e., those with no oocysts present and with oocysts present but no lysis agent, were included in every set of experiments (no definitive sign of cell lysis was observed; Ct values were >40). As an additional negative control, the Ag NP dispersant (NM-300 DIS) was added to oocysts, confirming that there was no impact of the dispersant on oocyst integrity (Ct values were >40). No negative controls are shown in the figures as the Ct values always exceeded 40. Triplicate samples were run, with two repeats performed on different days (n = 6). 

## 3. Results 

### 3.1. Exposure Time 

**Figure 1:**
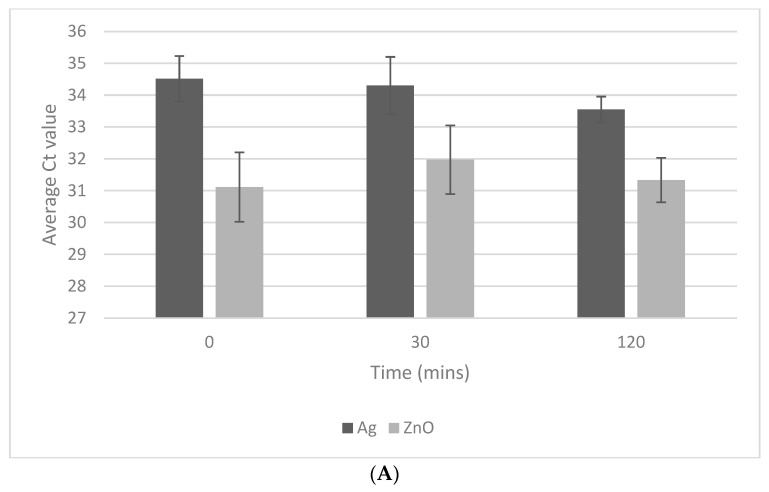

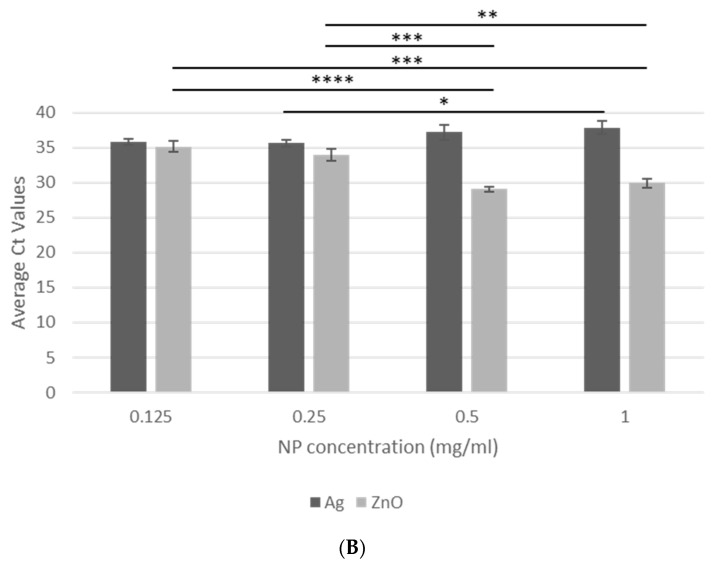
Optimising conditions for NP lysis. (**A**) Results from the PCR experiments comparing the performance of Ag and ZnO NPs at a range of different exposure times between 0 and 120 min. The graph plots Ct value against time at an oocyst number of 1000. No statistically significant differences were observed between the different time points (see below for statistical analysis information). (**B**) Results from the PCR experiments comparing the performance of Ag NPs and ZnO NPs at a range of NP concentrations between 0.125 and 1 mg/mL. The graph plots the Ct value against oocyst number in the sample. Negative controls resulted in Ct values in excess of 40 and they are not plotted. All data are expressed as mean ± standard error of the mean (SEM). For each endpoint, triplicate samples were utilised, along with technical replicates in the PCR. In addition, the experiments were repeated twice on separate days (total n = 6). Statistical analyses were performed using MATLAB (Mathworks, Natick, MA, USA). Data were analysed using one-way ANOVA with post hoc Tukey assessments. Significant difference *p* values are as follows: * *p* = 0.05; ** *p* = 0.01; *** *p* = 0.001; **** *p* = 0.0001.

We studied NP exposure times of 0, 30 and 120 min, followed by the DNA extraction and the purification protocol, and no statistically significant changes were noted between any of these times for either of the NPs (Figure 1A). 

### 3.2. NP Concentration 

NP concentration studies were then undertaken, adding the NPs to the sample and directly commencing the proteinase K incubation. The data indicate that increasing the concentration of Ag NPs resulted in less effective disruption of the oocyst wall (Figure 1B), with the only statistically significant difference being the higher Ct value at 1 mg/mL compared to the Ct value at 0.250 mg/mL. The opposite trend was observed for ZnO NPs (Figure 1B). For ZnO NPs, the average Ct values ranged from 35.1 for 0.125 mg/mL down to 29.0 for 0.5 mg/mL, with the Ct values for 0.125 mg/mL and 0.25 mg/mL being statistically significantly higher than those for both 0.5 mg/mL and 1 mg/mL. Dynamic Light Scattering data (Table 1) indicate that increasing NP concentration corresponds to a larger agglomerate size.

### 3.3. Oocyst Concentration 

**Figure 2:**
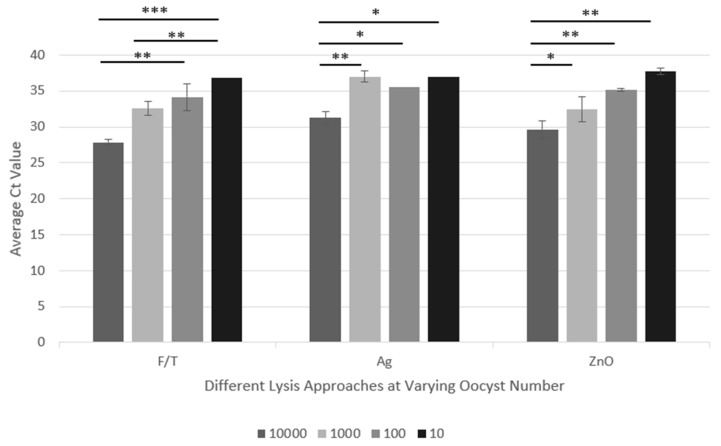
Oocyst detection. Results from the PCR experiments comparing the performance of Ag and ZnO NPs at a range of oocyst concentrations benchmarked against F/T. The graph plots Ct value against oocyst number in the sample. Statistical analyses and controls are as described in the Figure 1 legend (n = 6). Significant difference *p* values are as follows: * *p* = 0.05; ** *p* = 0.01; *** *p* = 0.001.

From Figure 1, optimal conditions were selected to investigate the NP performance over the range of 10–10,000 oocysts (Figure 2) and benchmarked against the freeze–thaw method (F/T). Figure 2 shows the expected linear relationship between oocyst number and Ct value (~3 Ct shift per 10-fold oocyst concentration) for the ZnO NP and F/T treatments (R2 > 0.9), though not for Ag NP. At all oocyst concentrations, there was no statistical difference in the Ct values for the ZnO NP (nor Ag NP, though as noted below use of these NPs did not give a linear relationship of Ct value with oocyst number) and F/T lysis approaches, indicating the ZnO NP approach is as effective as the commonly used F/T method.

## 4. Discussion

This study shows that oocyst lysis can be achieved using NPs, which is the first demonstration of the use of NPs for this application. The most effective oocyst disruption and DNA extraction were achieved at concentrations of 0.25 mg/mL and 0.5 mg/mL, for Ag and ZnO NPs, respectively. 

Previous studies have shown higher toxicity to bacterial cells at higher concentrations of nanoparticles [36,37], which also aligns with the trend observed for ZnO NPs between 0.125 and 0.5 mg/mL. Increasing concentrations of Ag NPs resulted in less effective disruption of the oocyst wall. The enhanced agglomeration of NPs can reduce toxicity to cells in vitro [38] and Dynamic Light Scattering data indicate that an increasing AgNP concentration corresponds to a larger agglomerate size, which could explain these observations. Smaller particle size generally enhances toxicity [39,40,41]. However, larger ZnO NPs were more effective than the smaller Ag NPs, in contrast to previous research with other microorganisms [32,42], which might be due to their increased solubility [31]. Interestingly, previous research suggested that the AgNP toxicity to *Cryptosporidium* was driven by a combination of ion release and particle effects [22]. 

Oocysts could be detected at all oocyst concentrations tested (10–10,000), indicating that all approaches were able to lyse and extract DNA over this concentration range. Less reliable results were obtained at oocyst concentrations of 10, with some samples being undetermined, which could reflect the difficulties of accurately obtaining a single oocyst in a sample via a dilution series. The Ag NP results were more variable whereas ZnO and F/T both performed well, with no statistical difference between the data. 

Future studies are required to optimise the approach, e.g., explore other NPs and make adjustments to the extraction and purification protocol to determine the limit of detection possible and to enable comparison with other approaches such as bead beating. Performance verification with other protozoan species; other *Cryptosporidium* kits; and environmental, food, and veterinary oocyst samples (including stool samples), as well as integration into miniaturised systems [43], would also be useful.

## 5. Conclusions

In conclusion, we have shown that ZnO NPs offer a viable alternative to F/T in disrupting the oocyst wall to enable the extraction of DNA via molecular methods (AgNPs effectiveness is lower). NPs offer a rapid lysis approach with no additional exposure/processing time required before the initiation of DNA extraction and purification protocols and do not require any additional equipment, giving several advantages over more traditional lysis approaches. 

## Figures and Tables

**Table 1:** Dynamic Light Scattering (DLS) spectroscopy was used to assess the hydrodynamic diameter and zeta potential of Ag and ZnO NP suspensions.

NP Concentration (µg/mL)	Ag NPs			ZnO NPs		
	Z-Ave (nm)	Pdl	Zeta Potential (mV)	Z-Ave (nm)	Pdl	Zeta Potential (mV)
1	44.1	0.39	−14.1	305.5	0.37	−4.7
5	47.06	0.35	−6.3	369.9	0.42	−12.6
10	79.29	0.28	−7.5	396.1	0.35	−11.5
20	94.31	0.42	−15.2	747.1	0.38	−5.8
50	130.27	0.25	−11.3	887.1	0.49	11.9

## Data Availability

Data are available on request.

## Supplementary Materials

The following supporting information can be downloaded at: https://www.mdpi.com/article/10.3390/mps7050066/s1, Figure S1. Positive control study conducted by adding ZnO NPs and AgNPs directly into positive control of the PCR kit alongside a standard positive control (left) to observe the potential inhibitory effects. No significant differences were found (n = 3).
