# Supplementary material for: Nanoparticle Lysis of Cryptosporidium Oocysts
Source: Methods Protoc. 2024 Aug 23;7(5):66. doi: 10.3390/mps7050066 (PMC11417895; doi:10.3390/mps7050066)
Supplement: Supplementary file 1 [file mps-07-00066-s001.zip › mps-3086862-supplementary.pdf]

Supplementary Information

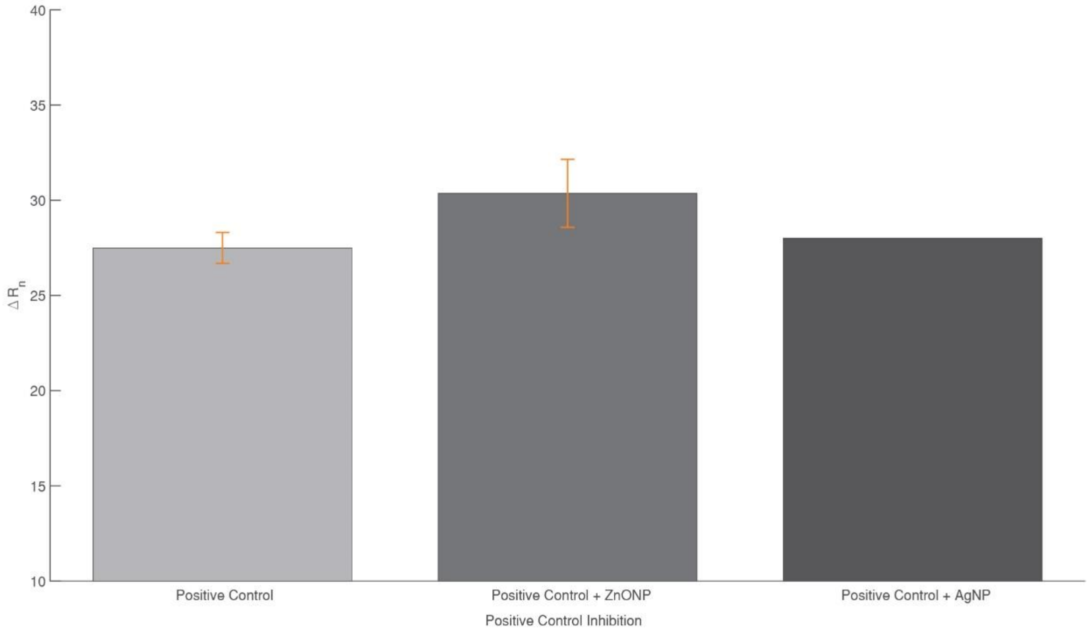

Figure S1. Positive control study conducted by adding ZnO NPs and AgNPs directly into positive control of the PCR kit alongside a standard positive control (left) to observe the potential inhibitory effects. No significant differences were found (n = 3).
